# Venous air embolism

**DOI:** 10.4103/0972-5229.60179

**Published:** 2009

**Authors:** Sourabh Aggarwal

**Affiliations:** University College of Medical Sciences, New Delhi, India

Dear Editor,

The article “U turn to venous air embolism”[1] by Singh *et al*, is quite interesting. I appreciate the authors for coming out with an indigenous method to prevent the life threatening complication of air embolism when a fluid infusion is complete and the drip set is still open. I would like to add my views to the same. It is worth mentioning that the “U turn” maneuver advocated by the authors should be set at a level lower than the lowest level to which arm, into which the infusion set is inserted, can reach by all probable means. It will ensure that the hydrostatic pressure in the “U turn” remains lower than that in the vein in the arm. The lower the “U turn” is set, the more negative intra thoracic pressure will be required to aspirate air, and thus, the chances will be reduced further. It is quite fascinating to observe that at this time of latest technological advancements, simple no-cost maneuvers can prevent such life threatening complications. I again applaud authors for their efforts.

## References

[1] Singh H, Tewari A, Kaur B, Garg S (2009). U turn to venous air embolism. Indian J Crit Care Med.

